# Nucleated synthetic cells with genetically driven intercompartment communication

**DOI:** 10.1073/pnas.2404790121

**Published:** 2024-08-26

**Authors:** Ion A. Ioannou, Carolina Monck, Francesca Ceroni, Nicholas J. Brooks, Marina K. Kuimova, Yuval Elani

**Affiliations:** ^a^Department of Chemistry, Imperial College London, Molecular Sciences Research Hub, London W12 0BZ, United Kingdom; ^b^Department of Chemical Engineering, Imperial College London, London SW7 2AZ, United Kingdom; ^c^fabriCELL, Imperial College London, London SW7 2AZ, United Kingdom; ^d^Imperial College Centre for Synthetic Biology, Imperial College London, London SW7 2AZ, United Kingdom

**Keywords:** artificial cells, membranes, cell-free protein expression, synthetic biology, vesicles

## Abstract

Eukaryotic cells are characterized by multiple chemically distinct compartments, one of the most notable being the nucleus. Within these compartments, there is a continuous exchange of information, chemicals, and signaling molecules, essential for coordinating and regulating cellular activities. One of the main goals of bottom–up synthetic biology is to enhance the complexity of synthetic cells by establishing functional compartmentalization. There is a need to mimic autonomous signaling between compartments, which in living cells, is often regulated at the genetic level within the nucleus. This advancement is key to unlocking the potential of synthetic cells as cell models and as microdevices in biotechnology. However, a technological bottleneck exists preventing the creation of synthetic cells with a defined nucleus-like compartment capable of genetically programmed intercompartment signaling events. Here, we present an approach for creating synthetic cells with distinct nucleus-like compartments that can encapsulate different biochemical mixtures in discrete compartments. Our system enables in situ protein expression of membrane proteins, enabling autonomous chemical communication between nuclear and cytoplasmic compartments, leading to downstream activation of enzymatic pathways within the cell.

## Significance

In living cells, architectural complexity through compartmentalization is closely linked with functional sophistication. To date, most synthetic cell systems are limited to single-compartment structures. When multiple compartments are present, encapsulation of complex mixtures is challenging, and intercompartment communication is usually absent. Here, we address this by developing an emulsion-based technology that allows us to create an inner compartment resembling a nucleus surrounded by a cytoplasm. Additionally, we can control the composition of both inner and outer compartments. This enables the encapsulation of distinct genetic material and biochemical pathways in different compartments, with signaling events between the two cellular regions mediated by the expression of membrane-bound machinery.

Compartmentalization is a fundamental feature of living cells, playing a crucial role in numerous biological processes. It enables the spatial segregation of cellular components, allowing for the partitioning of cellular functions into distinct regions or organelles (1, 2). Spatial segregation of content also allows cellular regions to exist out-of-equilibrium and to possess distinct chemical environments. Increased levels of compartmentalization support the coordination of complex biochemical processes and have been linked to enhanced metabolic efficiency and increased behavioral sophistication in cells (3, 4). Examples of compartmentalization within subcellular organelles include the segregation of genetic material in the nucleus, the generation of energy in mitochondria, the sorting of proteins in the endoplasmic reticulum (5–7), and regulation of cellular responses using membrane-less organelles such as stress granules (8, 9).

In recent years, there have been increasing efforts to replicate key architectural, functional, and behavioral aspects of biological cells (including compartmentalization) in synthetic ones. Synthetic cells (SynCells) are supramolecular biochemical systems constructed from the bottom–up, using biomolecular building blocks that are engineered to replicate key attributes of biological cells (10). They are increasingly used as models to understand the rules of life and as engineered microdevices in biotechnology, including through their use as therapeutic agents (11). Just as the evolution of compartmentalized systems contributed to increased behavioral complexity in living cells (12) (through the emergence of eukaryotic cells for example), compartmentalization can similarly be harnessed to create more functionally complex synthetic cells (13).

Mimicking the compartmentalized nature of cells by engineering synthetic ones with spatially segregated regions provides numerous advantages for the optimal function and regulation of cellular processes. Compartmentalization allows for spatial organization and segregation of (potentially opposing and incompatible) processes, reactions, and components within distinct regions, which helps prevent interference between the many coexisting pathways that exist in a cell (14). It also allows for division of labor and functional specialization by enabling compartments to exist in specialized physicochemical states that are optimized for specific biochemical processes (15–17). Efforts to build compartmentalized synthetic cells (18) span from enzymatic reaction-hosting vesicle-in-vesicle systems (19), to microfluidics-assembled vesosomes (20) and coacervate organelles encapsulated in liposomes (21). Compartmentalization has also served as the basis for stimuli-responsive activation and modulation of synthetic cell systems via compartment breakdown (22) and has been used to engineer complex biochemical processes into synthetic cells, including light-driven ATP synthesis that can power both DNA transcription and protein expression (23). There have also been efforts to dynamically reconfigure the compartments in response to chemical and mechanical cues (24, 25).

In subcellular organelles of living cells, there is constant crosstalk and exchange of signaling molecules, reaction intermediates, and biochemical products (26, 27). In many existing compartmentalized synthetic cells, however, compartmentalization is a static feature, with no exchange of materials between compartments. When exchange is present, this is typically mediated either by the passive diffusion of materials across membranes or by preinserted membrane proteins (20, 28–30). To unlock the full potential of synthetic cells as autonomous microdevices, there is a need to encode compartmentalization at the genetic level. In other words, communication between compartments should be under genetic control, and the exchange of contents and signaling between compartments should be the result of membrane machinery synthesized internally, driven by a genetic programme. This is possible since synthetic cells can be engineered to harbor synthetic genes which can be functionally linked to protein expression through transcription and translation systems (TX-TL) (31). To date, examples of genetically mediated communication between subcellular compartments are lacking. This is partly due to technological bottlenecks that have hindered the development of vesicle-in-vesicle subcompartment architectures, where the cargo of each compartment can be user-defined.

Herein, we have developed a methodology for creating cell-sized vesicle-in-vesicle assemblies (based on giant unilamellar vesicles, GUVs) as a means to mimic eukaryotic compartments, i.e., a “nucleus” within a “cytoplasm”, with the size of these compartments being comparable to those of eukaryotic cells (~ 5 to 30 µm diameter). This approach enables the encapsulation of distinct and predefined content within the nucleus and cytoplasm compartments. Communication between these compartments was genetically engineered by loading the nucleus with a genetic circuit encoding a membrane protein pore that was then expressed and integrated into the nuclear membrane. This resulted in chemical flux between compartments, triggering an enzymatic reaction in the cytoplasmic compartment.

## Results and Discussion

### Formation of Nucleated Giant Vesicles.

**Fig. 1.**
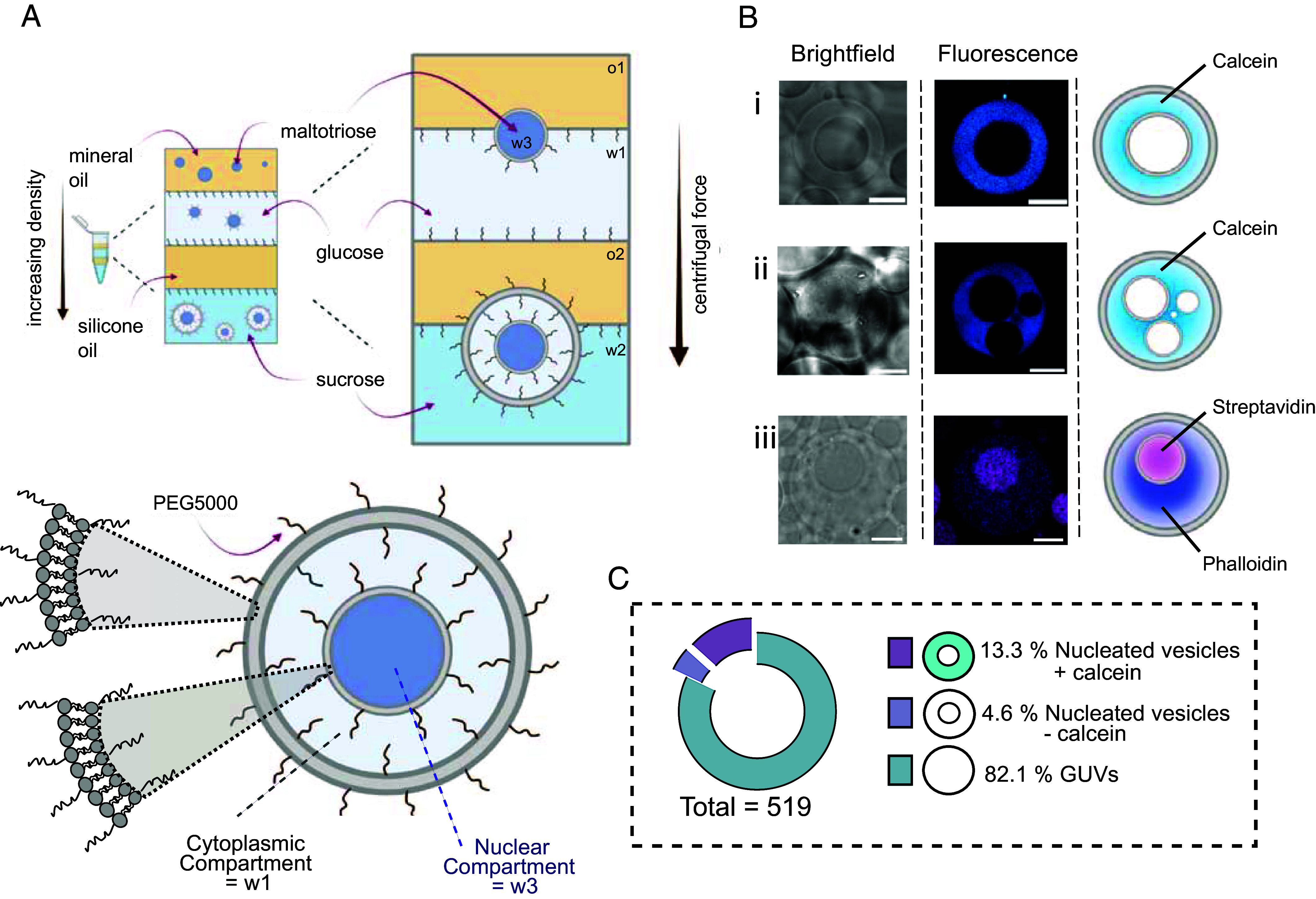
Generating a vesicle-in-vesicle: a nucleated vesicle. (*A*) Schematic of our method to generate nucleated vesicles via emulsion phase transfer. Water-in-mineral-oil emulsion droplets pass through an aqueous phase (glucose) that will eventually form the outer compartment after being centrifuged through a silicone oil layer. (*B*) i) Brightfield, fluorescence, and cartoon images of nucleated vesicles encapsulating calcein (cyan) in the outer compartment. ii) Examples of vesicles with several inner compartments containing calcein are occasionally observed. iii) Nucleated vesicles encapsulating biomolecules. Alexa488-labeled streptavidin (magenta) in the inner compartment and Alexa647-labeled phalloidin (cyan) in the outer compartment. (*C*) Overall yield of the various types of vesicles we generated. A total of 519 vesicles were analyzed (n > 3 independent repeats). For calcein and alexa488-labeled streptavidin, λ_ex_ = 496 nm, λ_em_ = 510 to 620 nm. For alexa647-labeled phalloidin, λ_ex_ = 633 nm, λ_em_ = 660 to 800 nm. w1 = 0.5 m glucose, w2 = 0.5 M sucrose, w3 = 0.4 M maltotriose, o1 = mineral oil, o2 = silicone oil (All scalebars are 10 µm.)

To enable genetically programmed communication between compartments, we first devised a method to create a vesicle within a vesicle, akin to a nucleus within a synthetic cell. A critical design criterion (not achievable using conventional methods) (32) was ensuring distinct and predefined compositions for the “nucleus” and “cytoplasm” compartments. Moreover, the dimensions of the inner compartment needed to be of a similar size regime to the external one but slightly smaller, mimicking a natural nucleus (33). While several methods exist for encapsulating vesicles within vesicles (32), these typically involve nanosized (c. 100 nm) vesicles for the inner compartments or rely on highly intricate microfluidic devices for assembly (20). To address these limitations, we developed a methodology (Fig. 1*A*) which builds on the emulsion phase transfer technique used in generating GUVs (34).

To generate vesicles-in vesicles, we used a system described by Ip *et al.* as a starting point (35). We assembled a multilayered column consisting of four oil/water/oil/water layers (from top to bottom: o1, w1, o2, w2), with lipids dissolved in the oil phases. Lipids self-assembled into a monolayer at each water/oil interface. To ensure column stability, the density of each upper layer had to be greater than that of the layer below. We achieved this by using oils with varying densities (o1 = mineral oil; o2 = silicone oil) and dissolving different sugars in the water phases (w1 = 0.5 M glucose; w2 = 0.5 M sucrose). Subsequently, a water/oil (o1, w3) emulsion was created (w3 = 0.4 M maltotriose) and deposited at the top of the column. Since w3 was denser than the other phases, centrifugation pulled the emulsion through the column. As the droplets passed through each water/oil interface, they acquired a lipid monolayer. At the column’s base, vesicles-in-vesicles formed, with the inner nuclear compartments comprising material from w3, the outer cytoplasmic compartment from w1, with the external phase being w2. In a departure from previous methods (35), we incorporated PEGylated lipids into the lipid composition. This led to an increase of at least 20× of multicompartment vesicles (from 0.8% without PEGylated lipids to 17% with PEGylated lipids, >300 vesicles analyzed). We hypothesize that use of PEGylated lipid introduces a steric barrier, preventing direct membrane-to-membrane interactions (shown in other related systems) (36) that lead to the formation of multilamellar vesicles through layer-by-layer assembly (35). Instead, our approach favored the generation of a vesicle-in-vesicle architecture, where membrane layers do not deposit directly upon one another. Importantly, our approach allowed us to easily load different materials in different compartments.

In order to visualize the successful generation of these nucleated giant vesicles, we encapsulated the fluorescent dye calcein (0.5 mM) in the glucose aqueous phase (w1), which resulted in a distinct ring-shaped morphology (Fig. 1 *B*, *i*) under epifluorescence microscopy. The ratio of produced nucleated vesicles was calculated to be 18 % (of total vesicle number), of which 74 % contained calcein in the outer ‘cytoplasmic’ glucose phase (Fig. 1*C* and *SI Appendix*, Fig. S1). More complex architectures with several internal compartments were also occasionally observed (ca. 2% of the total vesicle population; Fig. 1 *B*, *ii*). The produced vesicles were stable for at least 24 h, and no significant calcein leakage was observed after one day of storage at 4 °C. Finally, we demonstrated the ability to encapsulate larger biomolecules in both compartments, by loading the outer compartment with alexa647-labeled phalloidin and the inner compartment with alexa488-labeled streptavidin (Fig. 1 *B*, *iii* and *SI Appendix*, Fig. S2).

To further validate the proposed mechanisms through which we obtain our final vesicle-in-vesicle product, we recorded brightfield and fluorescence microscopy images of the emulsion structures at various points of the o/w/o/w column (*SI Appendix*, Fig. S3). In the top oil phase, we observed nonfluorescent water-in-oil droplets, which will later become the inner compartment of the nucleated vesicle. As the vesicles descend into the first aqueous phase (containing calcein), they transform into vesicles. The interior of these vesicles is nonfluorescent, indicating the presence of a lipid membrane that is impermeable to calcein dye. The vesicles then descend into another oil phase. As expected, we observed monolayer-coated water-in-oil droplets with vesicles inside. Importantly, the dye is retained in the middle compartment. Finally, as the droplets descend into the bottom aqueous layer, they transform into vesicles, resulting in the generation of vesicle-in-vesicle structures, i.e., nucleated vesicles, with different materials loaded in the inner and outer compartments.

### Genetically Encoded Communication in Nucleated Synthetic Cells: System Overview.

Developing a method for generating nucleated vesicles enabled us to increase their complexity and transform them into nucleated synthetic cells (nSynCells) and engineer genetically driven intercompartment communication by facilitating material transfer through protein pores expressed in situ. We choose to express the membrane pore protein α-hemolysin (α-HL) to transduce a chemical signal between the inner and outer compartments. α-HL is a well-characterized membrane pore that is often used to facilitate chemical communication through unilamellar lipid membranes (37–39). The monomers can insert into lipid membranes without the need for chaperones or complex reconstitution protocols, and self-assemble into functional heptameric protein pores, making α-HL extremely useful for autonomous initiation of communication. Encapsulated transcription and translation (TX-TL) systems for cell-free protein synthesis allowed the in situ expression and integration of α-HL into the internal vesicle membrane without forming pores in the external membrane. Cell-free expression was achieved using a DNA template containing the α-HL gene and the PURExpress TX-TL system, a commercially available system containing all purified biomolecular components needed for protein expression (40).

**Fig. 2.**
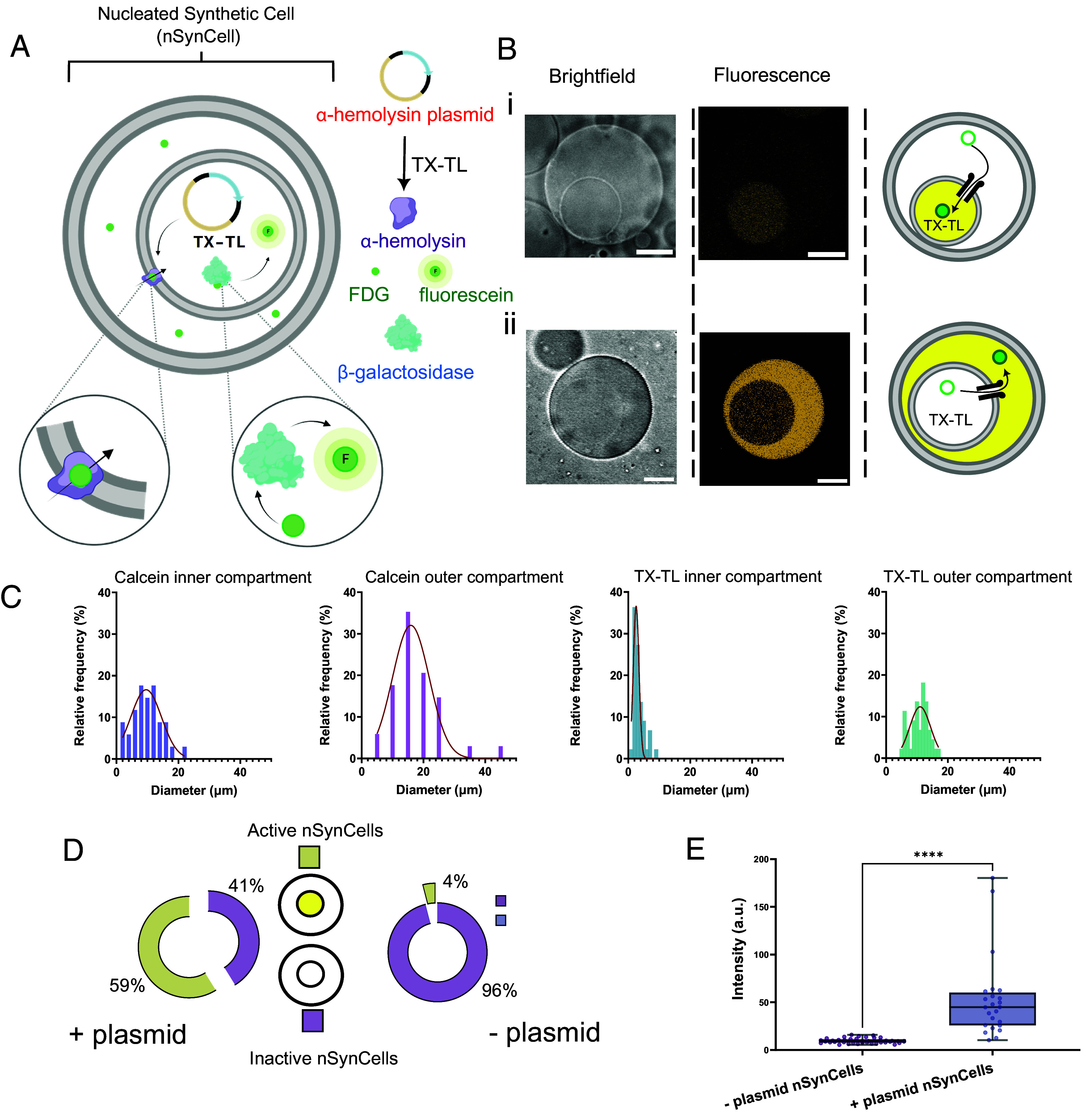
Nucleated SynCells with genetically programmed intercompartment communication. (*A*) Schematic of our experimental system. Transcription, translation, and incorporation of α-HL into the membrane of the inner compartment results in FDG influx and conversion to fluorescein by β-galactosidase. (*B*) Representative brightfield and fluorescence images of functional nucleated SynCells (nSynCells). *i*) In the first configuration, FDG is in the cytoplasmic compartment, passes through pores synthesized in situ, and gets converted to fluorescein in the nucleus. ii) In the second configuration, FDG is in the nuclear compartment and is converted to fluorescein in the cytoplasm. (*C*) Histograms presenting the relative frequencies of the diameters of the inner and outer compartments of nucleated vesicles encapsulating calcein and nSynCells containing TX-TL. (*D*) Proportion of nSynCells containing fluorescein in the inner compartment (active nSynCells) and nSynCells not containing fluorescein (inactive nSynCells) in the first configuration. In the left chart, α-HL plasmids were present in system; the right chart shows the control, where plasmids were absent. (*E*) Fluorescence intensity of nSynCells encapsulating the α-HL plasmid in the inner compartment was significantly higher than nSynCells without the plasmid, indicating that the expression and incorporation of α-HL into the inner membrane facilitated FDG transportation and the subsequent conversion to fluorescein from β-galactosidase. For fluorescein, λ_ex_ = 496 nm, λ_em_ = 510 to 620 nm (All scalebars are 10 µm.)

To engineer intercompartment signaling and communication in our nSynCells, we designed a system which allowed in situ expression of α-HL in the inner nuclear compartment, which then facilitated the movement of molecules between compartments, triggering downstream enzymatic reactions in localized SynCell regions. A schematic of our engineered intercompartment communication system is shown in Fig. 2*A*. In our system, we used a model enzymatic reaction involving the hydrolytic conversion of non-fluorescent substrate fluorescein-di-beta-D-galactopyranoside (FDG) into the fluorescent molecule fluorescein by the enzyme β-galactosidase (β-gal) (41).

We first engineered a nSynCell system where we encapsulate FDG in the cytoplasm, and a TX-TL system enriched with beta-galactosidase (β-gal) in the nucleus. In the absence of any plasmids, the SynCell system was expected to remain inactive with no fluorescent signal present. This is because the inner membrane acts as a barrier, preventing the membrane-impermeable substrate from interacting with the enzyme. However, the presence of the α-HL DNA template in the nuclear compartment is expected to activate the system. This should lead to the synthesis and insertion of α-HL, allowing the flow of FDG into the interior, followed by its enzymatic degradation and the evolution of a fluorescent signal in the internal vesicles. Thus, a fluorescent inner compartment could serve as a proxy reporter for α-HL expression and the subsequent transportation of FDG into the inner compartment (Fig. 2*A*). In all our experiments, nSynCells were formed and incubated at 37 °C in a polydimethylsiloxane (PDMS) chamber on a microscope slide with a coverslip using a heating plate for one hour to allow protein expression, before being imaged on a confocal microscope.

### Verification of Enzymatic Activity and Characterization of nSynCells.

At its narrowest, the β-galactosidase enzyme is 8.7 nm (42) in size, making it unable to pass through the α-HL pore, which has a minimum diameter of c. 1.5 nm (43). Consequently, any fluorescence in the internal compartment can only occur from the internal translocation of FDG. Following incubation, nSynCells containing the α-HL gene were seen to have a fluorescent nucleus (Fig. 2 *B*, *i* and *SI Appendix*, Fig. S4), confirming successful catalysis of FDG following expression and insertion of α-HL pores. We also performed nSynCell experiments where the compartment compositions were reversed, where plasmids, TX-TL, and β-gal were present in the outer compartment and FDG in the inner one. As expected, these experiments resulted in a fluorescent external compartment, with FDG flowing out of the nucleus following pore expression and insertion (Fig. 2 *B*, *ii*). With regard to the yield, similarly to calcein-containing constructs, 17% of SynCells (n = 99) had an inner compartment, of which 59% had a detectable fluorescein signal (Fig. 2*D*). In contrast, only 4% of the nSynCells without an α-HL DNA template (no expression of pores to facilitate FDG crossing the membrane) had inner compartments with higher fluorescence intensity. In this case, FDG permeation could be explained by transient poration of the bilayer due to osmotic imbalance (44, 45). To further assess this finding, we compared the intensities of all inner compartments with and without α-HL DNA templates. nSynCells expressing α-HL presented a sixfold increase in fluorescein signal than the ones lacking the DNA template, with mean values of 52.8 and 9.6 A.U., respectively (Fig. 2*E* and *SI Appendix*, Fig. S5).

Further characterization revealed that, overall, the total yield of these protein-expressing nSynCells was lower than those containing calcein, suggesting that yield is directly affected by both size and complexity of the loaded cargo. By analyzing the size of inner and outer compartments, we also observed that nucleated vesicles encapsulating calcein were larger than nSynCells encapsulating TX-TL with mean diameters of 10 µm and 3 µm for the inner compartments and 17 µm and 10 µm for the outer compartments, respectively (Fig. 2*C* and *SI Appendix*, Fig. S6). These results suggest that the size, chemical complexity, and osmolarity of the encapsulated solution affect the diameters of both inner and outer compartments and that larger compartments are not favored in the presence of complex mixtures (e.g., those found in TX-TL systems), potentially due to the resulting osmotic imbalances (46). We also found that the presence of the TX-TL mixture in the vesicles did not affect the ratio of vesicles with internal compartments compared to those without (*SI Appendix*, Fig. S7).

To further verify the ability of our system to express α-HL, and to allow FDG permeation and subsequent conversion into fluorescein by β-gal, we also performed experiments in regular GUVs without internal compartments. In this case, the GUVs encapsulated the plasmid, TX-TL, and β-gal, and the external phase contained FDG. When α-HL was expressed, the majority of GUVs presented a strong fluorescein signal (*SI Appendix*, Fig. S8*A*), compared to samples without an α-HL template, where negligible or no fluorescein signal was detected (*SI Appendix*, Fig. S8*B*). Finally, timelapse imaging of fluorescein intensity inside GUVs presented a rapid increase over 2.5 h at 38 °C (*SI Appendix*, Fig. S9), implying enzymatic activity from β-galactosidase (47). Conversely, the signal from the external solution also increased, but at a significantly slower rate, due to the presence of external unencapsulated β-gal that originated from droplets that did not successfully convert into GUVs during the emulsion phase transfer process (48).

Finally, we performed additional control experiments to verify that protein expression occurred only in the intended compartment and not in both compartments, which could theoretically happen due to TX-TL components leaking through transient pores that may open during the vesicle generation process. We demonstrated this by showing that calcein does not get encapsulated in the unintended compartment to any significant degree and by performing experiments showing that even if some material transfer occurs between compartments, the dilution level of TX-TL machinery would be too high for successful protein expression (*SI Appendix*, Fig. S10).

## Conclusions

A fundamental aspect of biology is that form and function are interlinked: architectural complexity, such as compartmentalization, leads to functional sophistication. To date, the exploitation of these principles in synthetic cells has been lacking. One underlying cause is a technological bottleneck preventing the controlled generation of nucleus-like subcompartments within synthetic cells. A microfluidic solution to create nuclei-like structures in vesicles does exist (20), but these methods require extensive expertise and training, creating a barrier to entry, making it inaccessible for synthetic biology laboratories with no cleanrooms or extensive microfluidic know-how. Nonmicrofluidic research groups often face challenges in constructing and operating synthetic cell microfluidic devices with reproducibility and ease. These devices are known for their complexity, making optimization and consistent operation difficult. Here, we introduce a user-friendly approach for forming nucleated giant vesicles, allowing us to engineer sophisticated nucleated synthetic cells by leveraging genetic componentry, protein expression machinery, membrane proteins, intercompartment signaling, and enzymatic cascade reactions. One appealing aspect of our method is its accessibility; it can be implemented with basic molecular biology laboratory equipment, such as a benchtop centrifuge. This feature makes the method available to nonmicrofluidic groups, thereby expanding its user base. Additionally, operating a microfluidic device typically requires large volumes of reagent in the syringes (around 200 to 500 μL), which is problematic when using expensive materials like TX-TL. In contrast, our method requires only low starting volumes (20 μL).

Importantly, this technology not only allows us to create an inner, nuclear-like compartment, but also to control the composition of both the nucleus and cytoplasm, and to encapsulate DNA, cell-free expression systems, and enzymatic components within predefined compartments. This allows us to transform our nucleated vesicles into nucleated synthetic cells. This advancement enables us to build synthetic cells with genetically programmed intercompartment communication events with the synthesis of membrane-embedded machinery for the first time. This nucleated configuration also establishes a shielded and stable environment for gene expression and enzymatic reactions, independent of the outer compartment or external conditions.

Our ability to generate compartmentalized synthetic cells with precise control over the composition of compartment interiors sets the stage for designing more complex synthetic cells with enhanced functional and behavioral sophistication. This potential will be further realized by integrating the increasing repertoire of synthetic cell modules, including those based on functional biomembranes (49), membrane proteins (50), genetic circuits (51), and molecular machines not found in biology, such as DNA nanoassemblies (52, 53). Such advancements will allow us to construct more biomimetic systems that operate out of equilibrium (54), addressing the current limitations of our system, which reaches equilibrium over the course of the experiment. This limitation largely stems from relying solely on passive diffusion through membrane pores for internal communication. To advance our system, future work could involve integrating active transport processes that utilize protein pumps and transporters, along with chemical, thermal, and light-powered systems (23). Moreover, to move beyond the constitutive nature of our current system, more dynamic control mechanisms could be developed through genetic regulation or physicochemical strategies, leveraging existing toolkits for building stimuli-responsive synthetic cells (22, 55, 56).

This work also paves the way for the encapsulation of synthetic genomes that are protected from potentially chemically reactive species that we might want to colocalize within the synthetic cells. It will also enable the construction of more representative synthetic eukaryote model where transcription and translation are separated into distinct compartments. This separation will allow us to explore the underlying reasons for this compartmentalization in natural systems, which are not currently well understood (57).

## Methods

All standard chemicals were purchased from Sigma Aldrich and were used without further purification. Alexa657-phalloidin and Alexa488-streptavidin were purchased from Thermo scientific. All lipids were purchased from Avanti Polar Lipids: 1,2-dioleoyl-sn-glycero-3-phosphocholine (DOPC), 1,2-dioleoyl-sn-glycero-3-phosphoethanolamine-N-[methoxy(polyethylene glycol)-5000] (18:1 PEG 5000 PE). Lipid stock solutions were prepared by dissolving lipid powders in chloroform to a concentration of 25 mg ml^−1^ and were stored at −20 °C.

### Formation of Nucleated Giant Vesicles.

Nucleated giant vesicles were produced with an optimized version of the protocol for the formation of multilayered GUVs developed by Tsoi et al. (35). Unless otherwise stated, lipid films were composed of 96 mol% DOPC and 4 mol% 18:1 PEG 5000 PE. For each experiment, two lipid-in-oil mixtures, one with mineral oil and one with silicone oil, were prepared at final concentrations of 5 mg ml^−1^.

Lipid-in-oil mixtures were prepared by dissolving lipid films in either mineral or silicone oils and were gently heated and vortexed before being sonicated for 1 h at 50 °C. Heating and vortex were repeated until a clear mixture was obtained.

Next, a column composed of two aqueous and two oil phases was assembled as presented in Fig. 1*A* in an Eppendorf tube (1.5 mL). The process for forming nucleated vesicles without the cell-free expression system follows the same procedure as described for the system with it, as described in the *methods* section below.

### Cell-Free Protein Synthesis Encapsulation.

In situ protein synthesis was achieved using the PURExpress® In Vitro Protein Synthesis Kit (NEB)—a reconstituted CFPS kit. Samples were prepared according to the manufacturer’s instructions, using 250 ng plasmid DNA and 0.5 uL Murine RNAse inhibitor (NEB). Then, 10 uL maltotriose (0.4 M final concentration) was also added to increase the density of this inner GUV phase. Communication experiments included 1 uL β-galactosidase at a working concentration of 10 units/mL, otherwise nuclease-free water was added to complete the sample volume up to 30 uL. This formed the aqueous cargo for the internal compartment and was added to 300 uL lipid-in-oil to create the emulsion. To build the column for nucleated vesicle formation, we layered (from the bottom, up) 150 uL 0.5 M sucrose (w2), 350 uL lipid in silicon oil (o2), 400 uL glucose (w1) with 0.5 mM FDG, emulsion described above containing 300 uL lipid in mineral oil (o1), 25 uL CFPS, β-galactosidase (5 units), and maltotriose (0. 4 M). The column was spun on a benchtop centrifuge at 8,000×g for 40 min. The supernatant was removed from the spun tube, taking care not to disturb the pellet. The pellet was resuspended in 150 to 200 uL of 0.5 M sucrose and moved to polydimethylsiloxane wells on a coverslip. All samples were then incubated at 38 °C on a heating plate for an hour to allow protein expression/control for the effect of heating.

### Construction of α-Hemolysin Plasmid.

The α-hemolysin plasmid was constructed using restriction digest cloning to combine a plasmid vector which had a T7 promoter, with the α-hemolysin gene. The vector was synthesized by DNA 2.0 (CA, USA), containing an *E. coli* pJexpress 441 vector with T7 promoter and terminator sequences (Catalog number: FP-03-441). The α-hemolysin gene was obtained from the pT7-WT-H6 plasmid, kindly provided by the Bayley group, University of Oxford (58). The ligated final product was transformed into chemically competent DH5α *E. coli* which were plated on LB agar containing 100 ug ml^-1^ Ampicillin and incubated at 37 °C overnight. Plasmids were recovered using a Mini Prep kit (Qiagen) and correct clones were identified via sequencing.

### Confocal Microscopy.

Microscopy images were acquired with a SP5 II confocal fluorescence microscope (Leica Microsystems) using either a 20× (NA: 0.7) objective or a 63× water immersion objective (NA: 1.2) with correction collars. The image resolution was 256 × 256 pixels. Liposomes containing calcein, fluorescein, or Alexafluor-488 labeled streptavidin were excited at 496 nm using an Argon laser and emission was recorded between 510 and 620 nm. Alexa647-phalloidin was excited at 633 nm and emission was recorded between 660 and 800 nm. Liposome samples were visualized in a square 15 × 15 mm PDMS chamber on a 24 × 50 mm cover glass and sealed with an 18 × 18 mm coverslip. Image processing was performed with Fiji (ImageJ), and graphs were plotted and analyzed with Prism (GraphPad Software).

## Supplementary Material

Appendix 01 (PDF)

## Data Availability

All study data are included in the article and/or *SI Appendix*.
