# Supplementary material for: Nucleated synthetic cells with genetically driven intercompartment communication
Source: Proc Natl Acad Sci U S A. 2024 Aug 26;121(36):e2404790121. doi: 10.1073/pnas.2404790121 (PMC11388312; doi:10.1073/pnas.2404790121)
Supplement: Supplementary file 1 — Appendix 01 (PDF) [file pnas.2404790121.sapp.pdf]

Supplementary Information

Nucleated synthetic cells with genetically driven inter-compartment communication

Ion A. Ioannou[1,2,4], Carolina Monck [2,3,4], Francesca Ceroni [2,3], Nicholas J. Brooks[1,4], Marina K. Kuimova[1], Yuval Elani[2,3,4]*

1 Department of Chemistry, Imperial College London, Molecular Sciences Research Hub, London W12 0BZ

2 Department of Chemical Engineering, Imperial College London, South Kensington, London SW7 2AZ

3 Imperial College Centre for Synthetic Biology, Imperial College London, South Kensington, London SW7 2AZ

4 fabriCELL, Imperial College London, South Kensington, London SW7 2AZ

* Email: y.elani@imperial.ac.uk

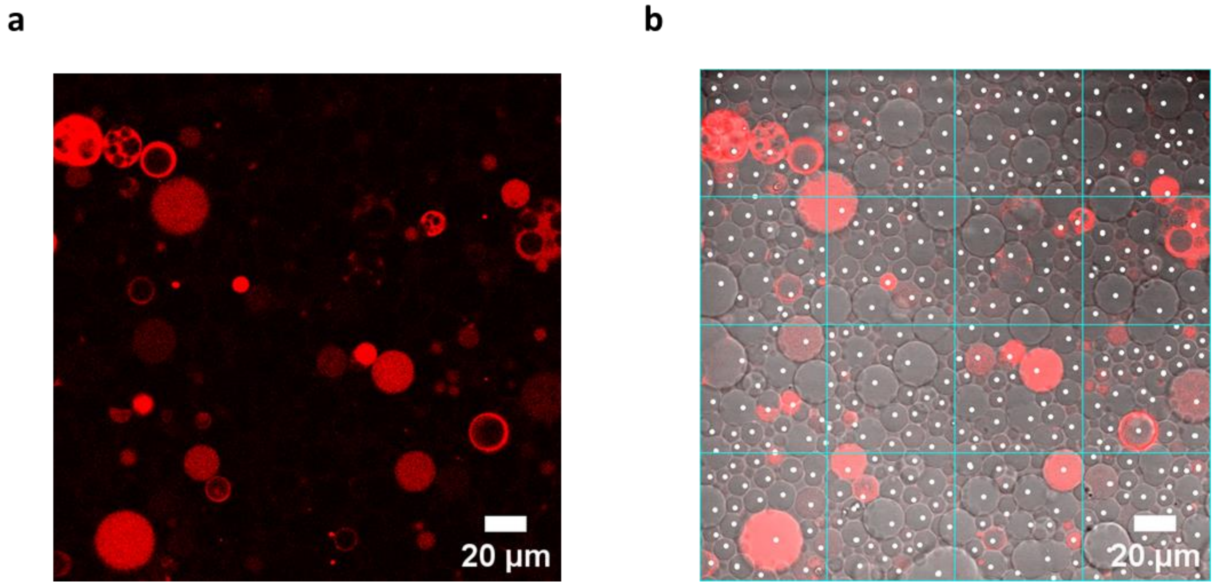

SI Figure 1. Analysis of nucleated vesicles. a) Fluorescence image of nucleated giant vesicles encapsulating calcein. Vesicles with an outer and inner compartment as well as multi-compartment vesicles can be distinguished. **b)** Overlap of brightfield and fluorescence images of a) used for the vesicle yield analysis. Every vesicle larger than 3 um was marked with a white dot. Analysis was performed with ImageJ (Fidji). λexc = 496 nm, λemis = 510-620 nm.

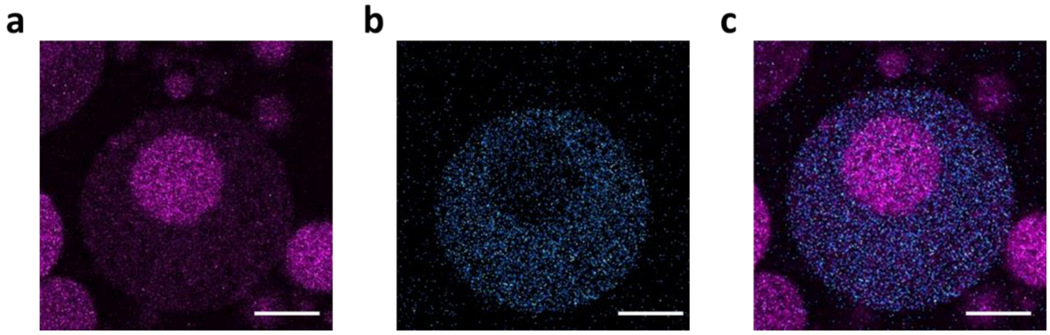

SI Figure 2. Encapsulation of different biomolecules in distinct compartments. a) Fluorescence channel of alexa488-streptavidin. λexc = 496 nm, λemis = 510-620 nm. **b)** Fluorescence channel of alexa647-phaloidin, λexc = 633 nm, λemis = 660-800 nm. **c)** Overlay image of the two fluorescence channels. Scalebars are 10 µm.

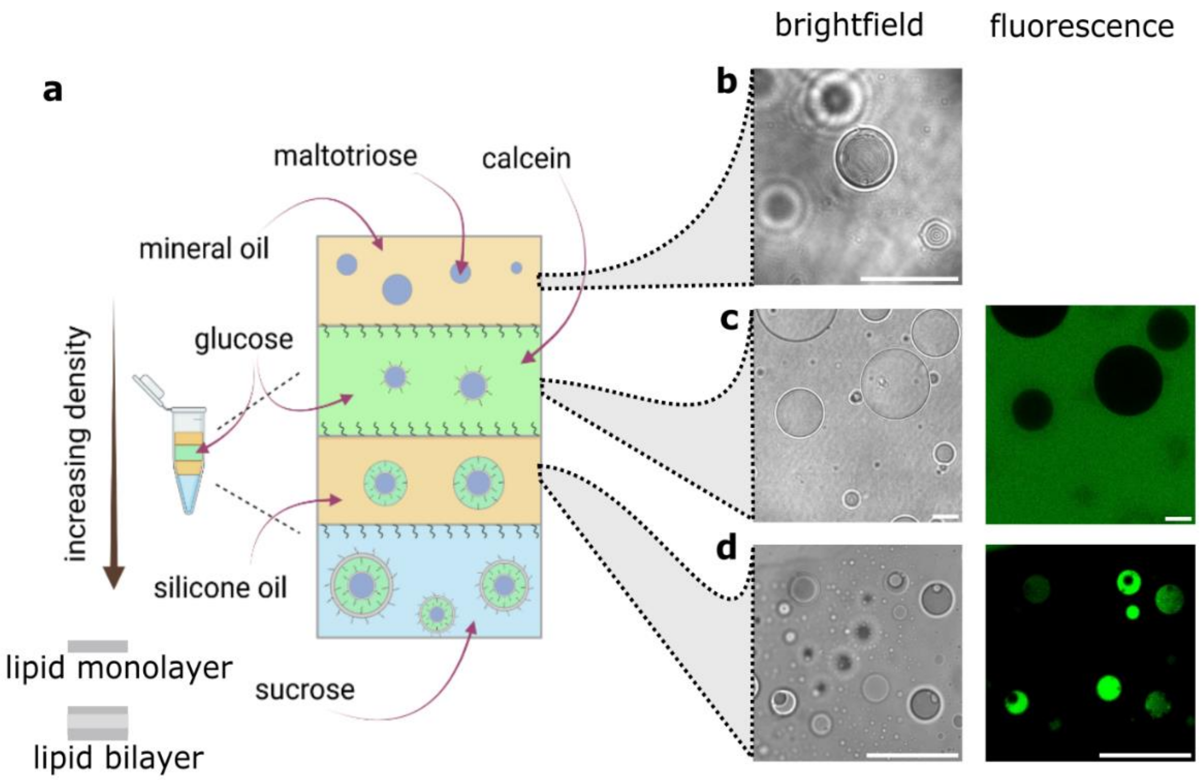

SI Figure 3. Capturing intermediate steps involved in nucleated vesicle assembly. a) Schematic illustrating the different intermediate stages of the emulsion phase transfer procedure, leading to the formation of nucleated vesicles as they descend to the bottom of the column. **b)** Microscopy images of lipid monolayer-coated water-in-oil emulsion droplets. **c)** Microscopy images of membrane-bound vesicles with calcein dye in the external solution. **d)** Microscopy images of monolayer-coated oil-in-water droplets containing single giant vesicles (nucleated vesicles) with calcein in the outer compartment. Scale bars = 20 µm.

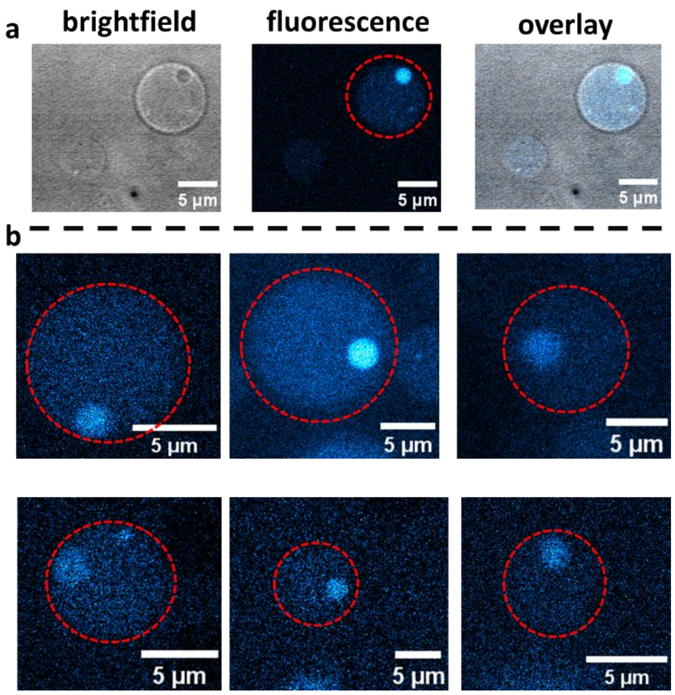

SI Figure 4. Representative images of nSynCells that have experienced inter-compartment communication events. a) Brightfield, fluorescence, and overlay images of nSynCells showing the accumulation of fluorescein signal in their inner compartment. This accumulation results from the transport of FDG from the outer compartment through α-HL into the inner compartment, where it interacts with β-galactosidase to produce the fluorescent product. **b)** Fluorescence images of several nSynCells that have experienced successful inter-compartment communication. Red dashed circles outline the periphery of the outer compartments. λexc = 496 nm, λemis = 510-620 nm

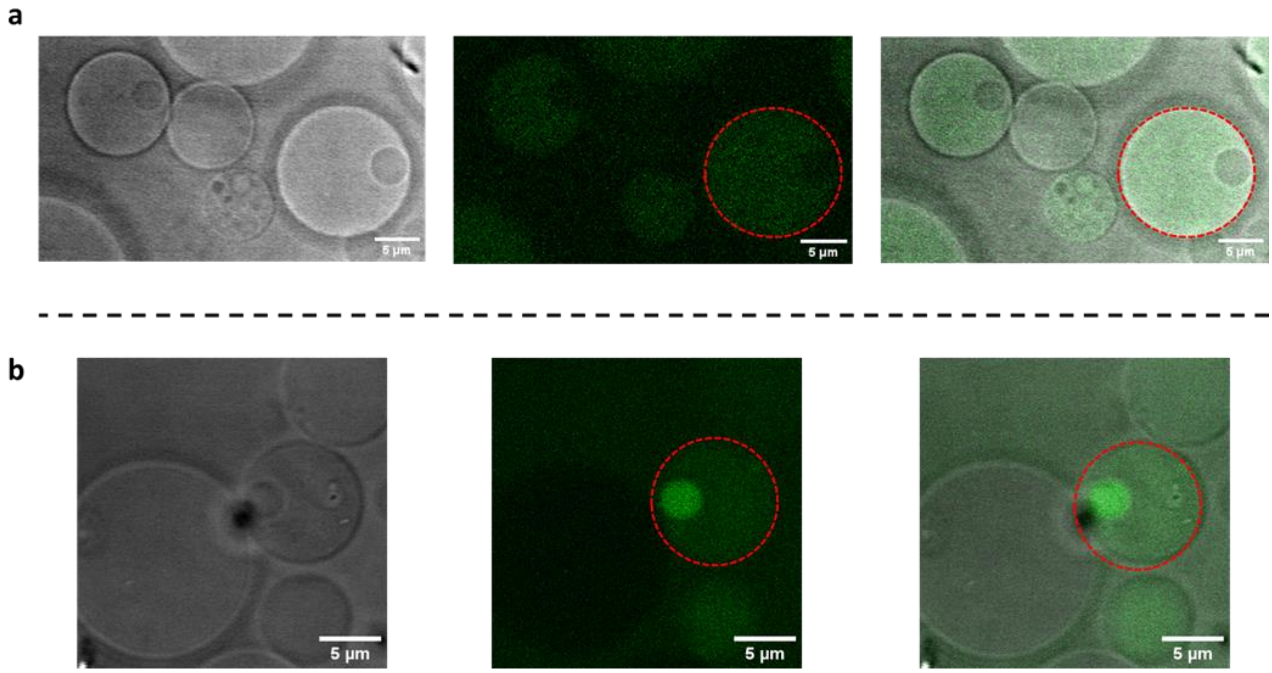

SI Figure 5. Representative images of nSynCells with and without α-HL plasmid. Brightfield, fluorescence and overlay confocal images of nSynCells **(a)** without α-HL plasmid (inactive) and **(b)** with α-HL plasmid (active). These nSynCells had nuclei of approximately the same mean fluorescence intensity values of Figure 2e (9.6 and 52.8 A.U. respectively) in their inner compartments. Red dashed circles outline the periphery of the outer compartments. λexc = 496 nm, λemis = 510-620 nm.

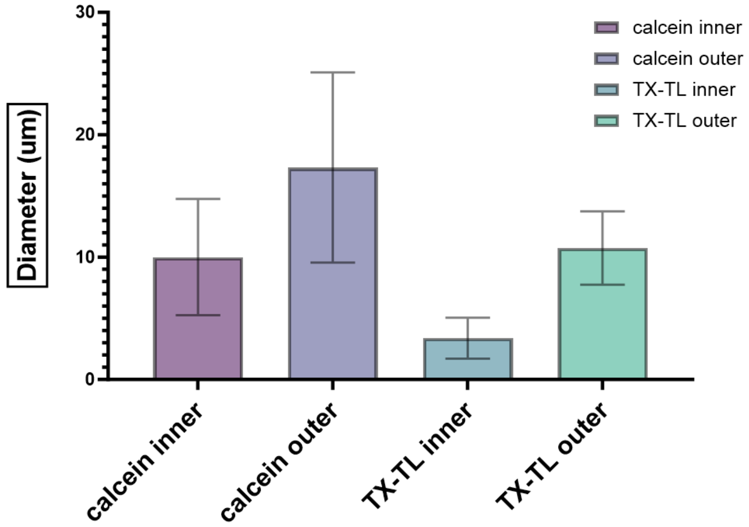

SI Figure 6. **Effect of the nature of encapsulated cargo on the size of nSynCell compartments.** Comparison of the inner and outer diameters of nucleated vesicles encapsulating calcein and nSynCells encapsulating TX-TL with β-galactosidase and the α-HL plasmid in the inner compartment. Mean values for calcein-containing nucleated cells are higher than the TX-TL ones, suggesting that the size and complexity of the encapsulated solution affect the diameters of both inner and outer compartments. Error bars are standard deviations.

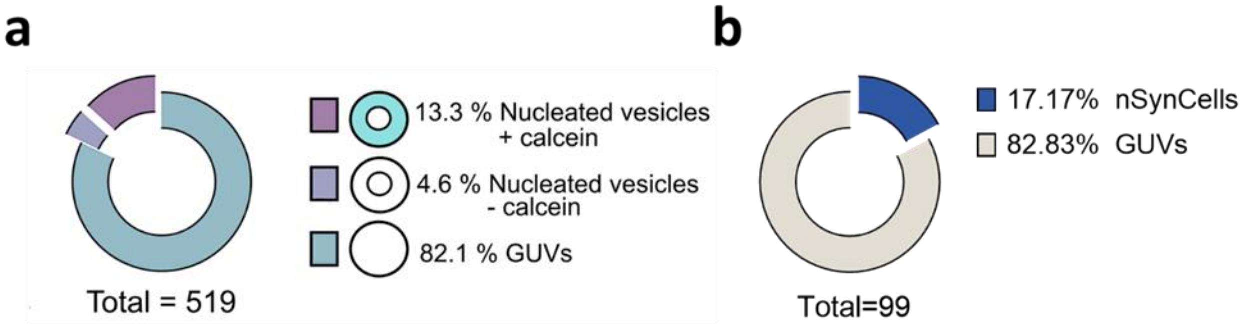

SI Figure *7***. Number of vesicles with an inner compartment in different conditions a)** 17.9% of the vesicles encapsulating calcein presented an inner compartment. A total of 519 vesicles were analysed (n>3 independent repeats). **b)** No significant difference was observed with the ratio of giant vesicles encapsulating the TX-TL and FDG/β-gal system calculated at 17.17%. A total of 99 vesicles were analysed (n=3 independent repeats).

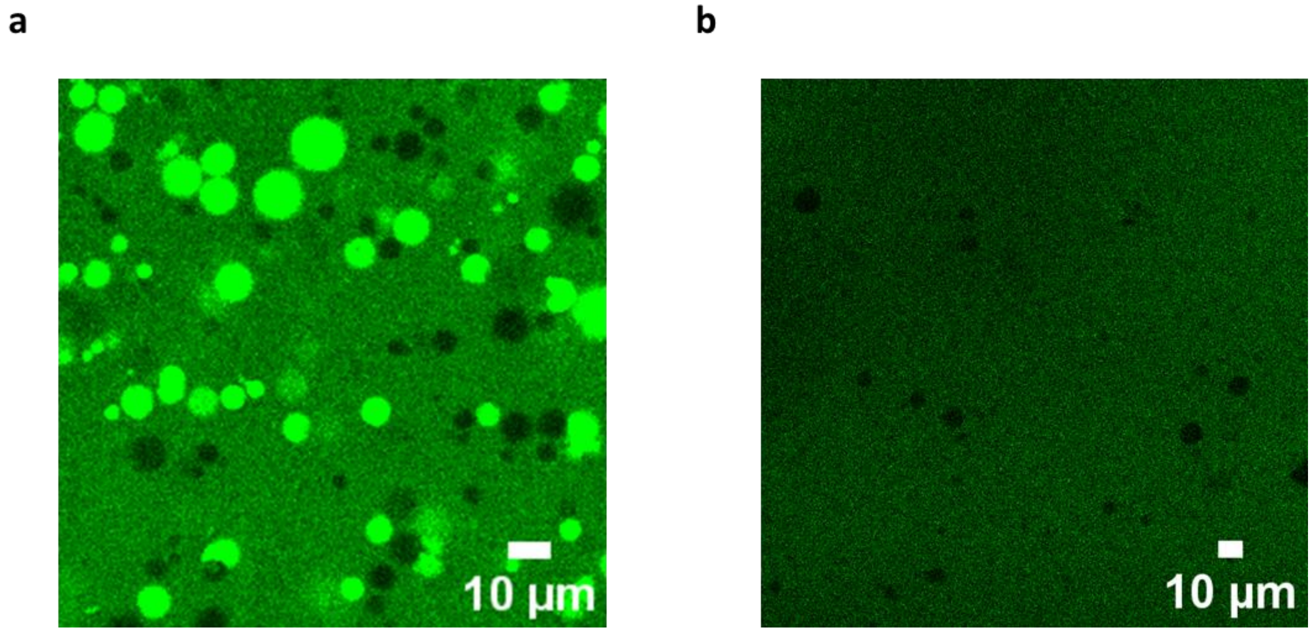

SI Figure 8. Verification of communication assay in GUVs. a) GUVs encapsulating the TX-TL kit with β-galactosidase and α-HL plasmid. After α-HL expression FDG from the outer solution enters the GUVs and is converted to fluorescein by β-galactosidase. TX-TL encapsulation is not homogeneous everywhere, resulting in dark GUVs where α-HL is not expressed. **b)** Without the presence of α-HL plasmid, the majority of GUVs did not express any fluorescein signal.

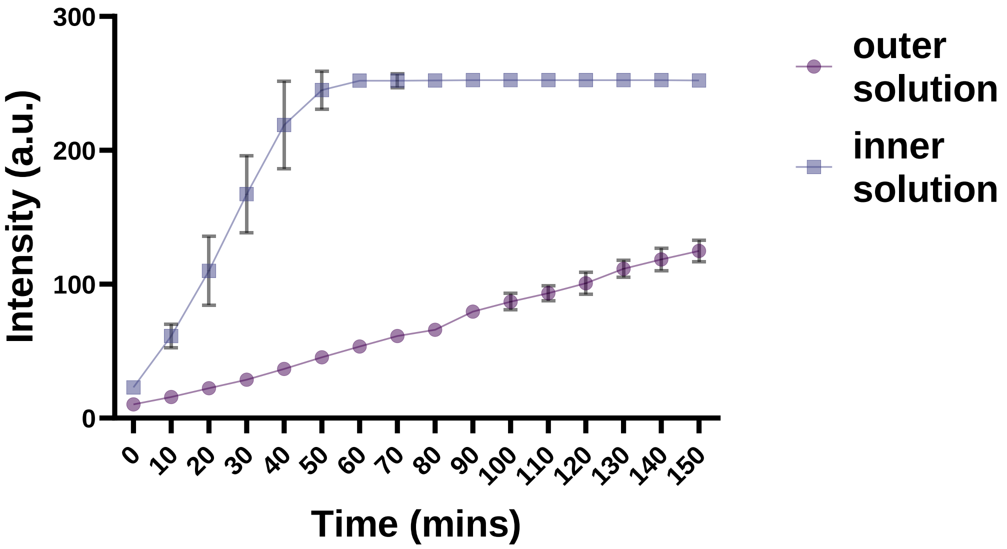

SI Figure 9. Fluorescence time-course of communication assay in GUVs. Evolution of fluorescent intensity for the experiment outlined in SI Figure 8, above. Fluorescence intensity graphs of GUVs encapsulated (inner) solution (TX-TL, α-HL plasmid, β-galactosidase) and outer solution (FDG). The inner solution's intensity increases exponentially due to the conversion of FDG to fluorescein from β-galactosidase as a result of expression and insertion of α-HL to the lipid membrane that allows FDG to enter the GUVs. The fluorescence intensity of the outer solution is linear and can be justified by unencapsulated β-galactosidase that ends up in the outer solution during phase transfer.

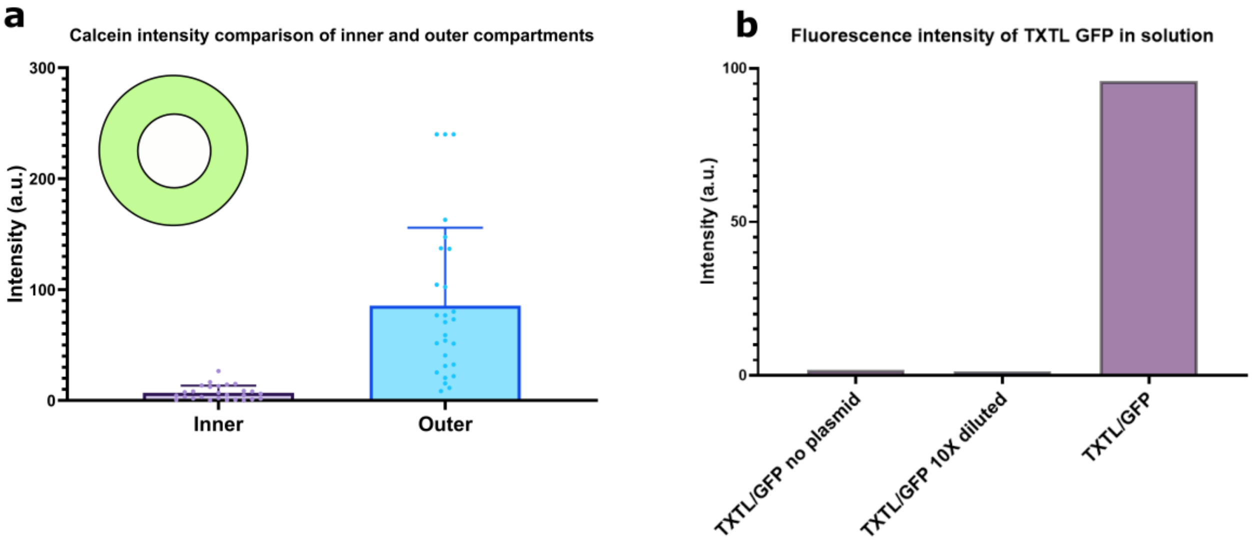

SI Figure 10. Control experiments to verify compartment-specific protein expression. **a)** Comparison of the fluorescence intensity between the inner and outer compartments obtained via confocal microscopy, where the nucleated vesicles were formed with 0.5 mM calcein in the outer compartment. A ~10-20 fold difference in mean fluorescence intensity was observed, indicating minimal inter-compartment leakage of content during the vesicle generation process. Data points represent individual vesicles, and the error bars correspond to standard deviation. **b)** Fluorescence intensity values obtained with a fluorimeter of a TX-TL system constitutively expressing GFP, showing that with a 10-fold dilution of reaction components, no GFP was produced. This leads us to conclude that even if there is minimal leakage of TX-TL components between compartments, it is not enough to lead to protein production in the unintended compartment.
